# Felons

**Published:** 1870-07

**Authors:** 


### FELONS.

There are two ways of curing felons promptly: 1st, Cut the finger off. 2d. Take a lancet and plunge it in, down to the very bone; make it grate on the bone so as to make sure that it is down far enough, because it is nothing more or less than a common boil formed under the ligament or whitleather, which is so tough that the matter cannot get out; if on the flesh, the boil gets well by breaking the skin, and thus pouring out its yellow matter, which was made by bad blood; hence all boils are curative and prevent serious disease.

There is a less objectionable cure for a felon, which may be known to be such by its "beating" for it is pulsationary; as soon as this beating is noticed, get a cantharides blister plaster, as large as the thumb-nail, apply it to the spot, let it remain six hours, when you will see the felon just under the skin, the core, as a core of a boil, which can be picked out with the point of a needle.

